# Challenges in diagnosis and biomarker testing for RET-altered lung and thyroid cancer care: an international mixed-method study

**DOI:** 10.1186/s12909-023-04396-w

**Published:** 2023-06-05

**Authors:** Patrice Lazure, Anthony Sireci, Vivek Subbiah, Suzanne Murray, Christian Grohé, Steven I. Sherman, Elizabeth Kelly, Patrick Bubach, Sophie Péloquin

**Affiliations:** 1 AXDEV Group Inc., 8, Place du Commerce, Suite 210, Brossard, Québec J4W 3H2 Canada; 2 Eli Lilly, New York, NY USA; 3 The University of Texas MD Anderson Cancer Center, Houston, TX USA; 4 Berlin Evangelical Lung Clinic, Berlin, Germany

**Keywords:** Lung cancers, Thyroid cancers, Medullary thyroid cancer (MTC), Pathology, Needs assessment, Diagnostic techniques and procedures, RET-alteration, Genetic biomarker testing, Quality assurance, Continuing professional development

## Abstract

**Background:**

The introduction of new targeted therapies for RET-altered lung and thyroid cancers (LC/TC) has impacted pathologists’ practice by making genomic testing more relevant. Variations in health systems and treatment access result in distinct clinical challenges and barriers. This study aimed to assess practice gaps and challenges experienced by pathologists involved in the diagnosis of RET-altered LC/TC, including biomarker testing, to inform educational solutions.

**Methods:**

Pathologists in Germany, Japan, the UK, and US participated in this ethics-approved mixed-methods study, which included interviews and surveys (data collected January-March 2020). Qualitative data was thematically analysed, quantitative data was analysed with chi-square and Kruskal–Wallis H-tests, and both were triangulated.

**Results:**

A total of 107 pathologists took part in this study. Knowledge gaps were reported regarding genomic testing for LC/TC in Japan (79/60%), the UK (73/66%), and the US (53/30%). Skill gaps were reported when selecting genomic biomarker tests to diagnose TC in Japan (79%), the UK (73%) and US (57%) and when performing specific biomarker tests, especially in Japan (82% for RET) and in the UK (75% for RET). Japanese participants (80%) reported uncertainty about what information to share with the multidisciplinary team to ensure optimal patient-centered care. At the time of data collection, pathologists in Japan faced access barriers to using RET biomarker tests: only 28% agreed that there are relevant RET genomic biomarker tests available in Japan, versus 67% to 90% in other countries.

**Conclusions:**

This study identified areas where pathologists need additional continuing professional development opportunities to enhance their competencies and better support delivery of care to patients with RET-altered lung or thyroid tumours. Addressing identified gaps and improving competencies of pathologists in this field should be emphasised in continuing medical education curricula and through quality improvement initiatives. Strategies deployed on an institutional and health system level should aim to improve interprofessional communication and genetic biomarker testing expertise.

## Introduction

The RET proto-oncogene codes for a transmembrane protein kinase can spur the growth of cancer cells when it is the activated product of a gene altered through gene fusion or mutation [1]. Although RET fusions are known to occur across cancer types, they are more frequently found in thyroid, and to a lesser degree, lung cancers [2]. Activating mutations in RET, however, are known to be oncogenic in medullary thyroid cancer (MTC) [3], making this RET-altered tumour different than papillary thyroid cancer or non-small-cell lung carcinoma (NSCLC). The prevalence of RET mutations in MTC is 25–60% [4, 5]. RET fusions are seen in fewer than 10% of papillary thyroid cancers [6] and approximately 2% [7] of NSCLC tumours, similar to the incidence of ROS1 fusions. Regardless of prevalence, targeted therapies that specifically target altered RET have shown considerable efficacy in patients with RET fusion-positive tumours [8].

Integration of genomic biomarker testing for RET-altered lung and thyroid cancers varies by country and clinical setting [9]. Currently, access to genomic biomarker testing depends on national guidelines, health system, reimbursement models in place, and patient profile [10]. The stage at which a patient is provided genomic biomarker testing for RET alterations (if available) can also depend on factors such as family history of multiple endocrine neoplasia (for patients with MTC), or history of smoking (for patients with NSCLC) [10, 11]. The tests performed depend on availability, approved therapies, and the prevalence of a given alteration [9, 12].

Pathologists have an essential role in shaping clinical practice for RET-altered cancers [13]. Though their exact role may vary by setting and country, the core responsibilities for preparing the tissue for testing (tissue triage), reporting, and interpretation are consistent across the discipline [14]. Pathologists (in the US this is done by anatomical pathologists specifically) identify the amount of tissue needed, appropriate tumour content, and best site on slide, depending on tumour histology for the selected molecular testing platform used [12]. Although pathologists’ presence at tumour boards is inconsistent [14], pathologists, when involved, can offer specialised insight into what biomarkers should be tested for and which tests should be ordered to ensure samples have been comprehensively assessed for all suspected alterations. In the US, for RET, tissue triage may be done by molecular pathologists specifically, who also engage with assay design, quality and validation. Pathologists are responsible for achieving accurate results through the selection of appropriate tests and methodologies, and for generating reports within a clinically-relevant timeframe [15]. By providing reports that have important implications on subsequent decisions made by a team of healthcare providers (in this case pulmonologists, endocrinologists, and medical oncologists) [16, 17], they have a distinct role in shaping clinical practice and making critical decisions, by identifying druggable biomarkers [18].

The clinical stage at which biomarker testing occurs varies by geography due to difference in government regulations and reimbursement. For example, in Japan RET testing is generally performed within the context of clinical trials for patients with NSCLC, though there are indications that this is changing [19–22] and recent ESMO guidelines suggest earlier testing in certain scenarios [9]. Timing and process for testing for RET alterations may also differ depending on the type of thyroid cancer. For example, MTC germline mutation testing is done earlier in the diagnostic process [9, 23] if hereditary RET mutations are suspected [9, 24]. As with lung cancer, the use of testing and available treatments varies by country [25].

Pathologists face challenges when contributing to the diagnosis of RET-altered thyroid and lung cancers. For example, studies show that biopsy practices often do not produce appropriate samples to allow for all of the workup and testing required for NSCLC [26]. Test panels do not all have the same comprehensiveness and do not always appropriately cover the full range of alterations seen in relevant biomarkers. There remains a reliance on and need for coordinating off-site testing and analysis [27]. Despite improvement in this space, challenges related to tissue procurement, tissue triage and appropriate test selection remain [12, 28].

There is a need to cultivate a broader awareness and understanding of which biomarker tests are currently available, as well as establish the evidence-based utility of biomarker testing for thyroid cancer in particular [29], an issue that may be improved by the establishment of a multidisciplinary team, as well as facilitating access to, and reimbursement for genomic testing [30].

A better understanding of these issues and those that may emerge from novel clinical practices, treatments, and innovations, are necessary to provide adequate education for pathologists involved in the testing and diagnosis of RET-altered lung and thyroid cancers.

This study was part of a broader research project designed to explore the care of patients with RET-altered lung or thyroid cancer, which also included the perspective of medical oncologists, pulmonologists, and endocrinologists. This article reports on the findings related to the following objective: to assess clinical practice gaps and challenges experienced by pathologists involved in RET-altered lung and thyroid cancer care in Germany, Japan, the United Kingdom, and the United States.

## Methods

This study used a mixed-method approach that combined semi-structured interviews with open-ended questions, to explore the in-depth challenges and experiences of the pathologists involved, and online surveys, to understand the current practical use of biomarker testing for RET-altered thyroid and lung cancers in the selected countries [31]. The qualitative and quantitative data collection phases were deployed in parallel between February and April 2020.

All components of this study were reviewed and approved by an independent ethical review board, VERITAS IRB (Quebec, Canada). All participants agreed to an informed consent form prior to study participation and those who completed the study received compensation based on the nature of their participation (interview or survey), their country of practice, and their profession, in alignment with fair market value and best ethical practices [32].

Recruitment was done using separate panels of healthcare providers which were both compliant with ESOMAR (European Society for Opinion and Marketing Research) guidelines [33]. Email invitations in each country’s main language (English, German, Japanese) included a secure link which led the participant to the screener and the informed consent form.

To be eligible to participate, pathologists needed to be in active practice (i.e., not retired or only in research or teaching), examine a minimum of 10 samples of lung or thyroid cancer a year, and have a minimum of three years of practice in either Germany, Japan, the UK, or the US. Maximum variation purposive sampling criteria were applied to ensure that a diversity of qualified participants and perspectives were included based on their region (state, province), community (urban, suburban, rural), gender, years of practice, and practice setting (i.e., academic or community-based) [34]. Enrolment was closely monitored by the research team, and specific sub-categories were closed if they were to reach a disproportionate portion of the final sample.

All data collection was conducted in each country’s main language. The qualitative interviews included open-ended questions regarding the challenges, practice gaps, and barriers that interviewees face in relation to their role in lung and thyroid cancer care. The quantitative survey was comprised of questions where participants reported their level of knowledge and skills on five-point scales (from none to expert), their level of agreement with specific statements using a five-point Likert-like scale, and the frequency with which they perform certain tasks (5-point scale, from never to frequently). Confidence was self-reported using a 0 to 100 visual analogue scale and participants indicated their continuing education preferences from a multiple-choice list of options.

NVivo software (QSR International Pty Ltd, Version 12, 2018) was used to perform a thematic analysis. Data from the qualitative phase was transcribed (and translated to English when required), coded, and organised into themes which were then reviewed and systematically analysed to identify patterns and meaningful insights into the experiences of pathologists working in the field [35].

Survey data was analysed using SPSS software (SPSS Statistics for Windows, version 27.0, 202, IBM Corporation, Armonk, NY), organised into frequency tables, and tested for frequency and variance using the chi-square and Kruskal–Wallis H tests, respectively [36]. For each survey question, participants were given the option to indicate that an item was “not relevant to my current practice” and, to avoid skewed data, these responses were excluded from the analysis. The 5-point Likert-like scale used for the knowledge and skill items was recoded into two categories: “sub-optimal” (indicative of a gap) when survey participants selected 1–3 (1-low, 2-basic, 3-intermediate) out of 5, and “optimal” when participants selected 4 or 5 (4-advanced, 5-expert). The 5-point Likert-like scale used for the agreement items was recoded into 3 categories: “disagree or strongly disagree,” “neither agree nor disagree,” and “agree or strongly agree.”

All analysed data was then triangulated to identify common and complimentary themes that emerged from both phases of the study and from the review of the literature [37]. These findings were interpreted collaboratively between educational experts [including co-authors PL, SM, SP] and clinical subject matter experts [co-authors AS, VS, CG, SIS].

## Results

A total of 16 interviews and 91 surveys were completed by clinical pathologists as a part of a broader study that also involved an additional 44 interviews and 440 surveys with pulmonologists and endocrinologists in the four targeted countries. Medical oncologists were included in all countries except Japan, where it is considered an emerging profession – lung and thyroid cancers are treated mainly by pulmonologists and endocrinologists [38]. Pathologist participant demographics varied, though the majority were from academic practice settings and had between 11 and 20 years of experience (see Tables 1 and 2). While data from pathologists were the focus of this analysis, data from all other profession/specialty groups were used for triangulation purposes, especially for findings related to team communications and multidisciplinary collaboration.

**Table 1:** Pathologists by country, practice setting and years of practice

	**Interviews** (*n* = 16)	**Surveys** (*n* = 91)	
**Country**			**Total (*n* = 107)**
Germany	4	20	24
Japan	4	30	34
UK	4	11	15
USA	4	30	34
**Years of Practice**			**Total**
3–10 years	25%, 4	29%, 26	28%, 30
11–20 years	50%, 8	47%, 43	48%, 51
21 + years	25%, 4	24%, 22	24%, 26
**Practice Setting**			**Total**
Academic Hospital	56%, 9	52%, 47	52%, 56
Community^a^	13%, 2	35%, 32	32%, 34
Multi- or single-specialty physician group practice	-	8%, 7	7%, 7
Specialised cancer center	31%, 5	3%, 3	7%, 8
Other	-	2%, 2	2%, 2

^a^Community hospital, community clinic, government medicine (e.g., veteran’s affairs)

**Table 2:** Non-pathologist participants by specialty and country

	**Country**	**Interviews (*n* = 44)**	**Surveys (*n* = 444)**	**Total**
**Medical oncologists**	Germany	4	44	145
	Japan	-	-	
	UK	4	44	
	USA	4	45	
**Pulmonologists**	Germany	4	36	169
	Japan	4	44	
	UK	4	28	
	USA	4	45	
**Endocrinologists**	Germany	4	26	174
	Japan	4	43	
	UK	4	45	
	USA	4	44	

In relation to pathology, this study identified challenges related to four areas: 1) selecting, performing, and interpreting biomarker tests, including those specific to RET; 2) communication and collaboration structures to support decision-making; 3) perception of clinical trials for RET inhibitors; and 4) barriers associated with government regulations on treatments and genomic testing for lung and thyroid cancers. Verbatim quotes presented in this section are representative of the most substantive themes that emerged from the qualitative analysis.

1. Challenges in selecting, performing, and interpreting biomarker tests

**Fig. 1:**
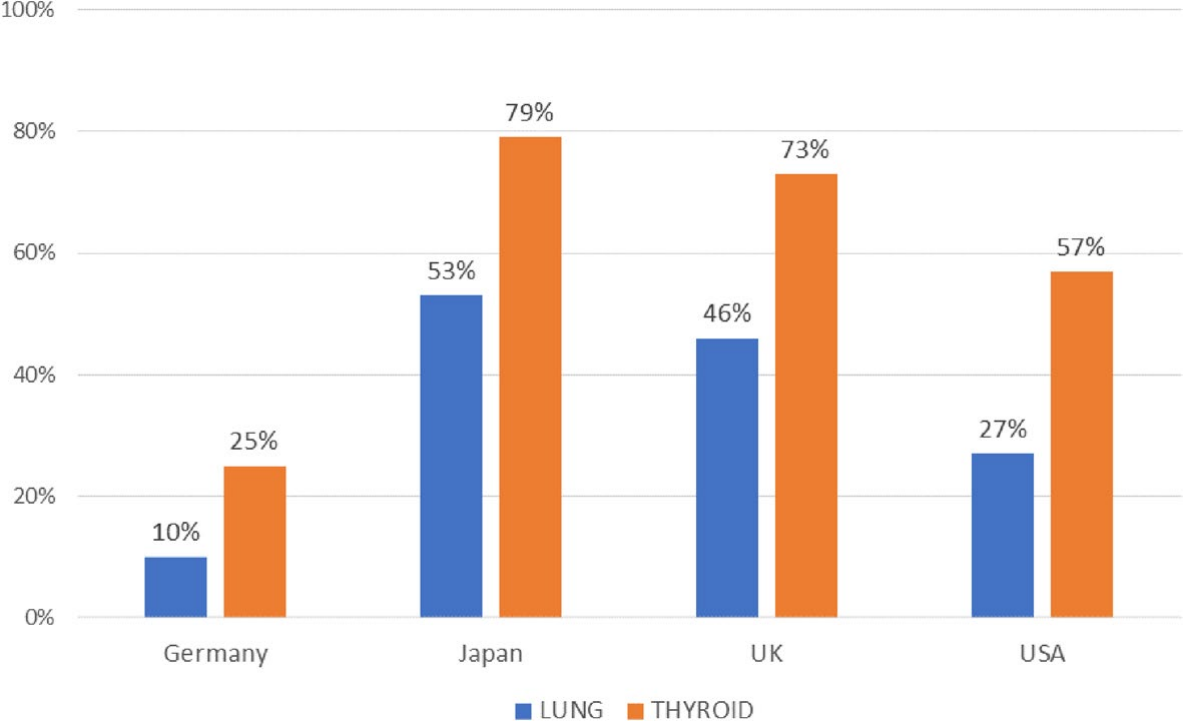
% Pathologists with sub-optimal skill ordering / selecting genomic biomarker tests to diagnose lung & thyroid cancer by country

Pathologists indicated sub-optimal knowledge in selecting appropriate genomic biomarker tests for thyroid (*p* = 0.001) and lung cancers (*p* = 0.009) in Japan (79% and 60% respectively), the UK (73% and 55%), and the US (53% and 30%) (Table 3). A lower proportion of German pathologists reported sub-optimal skills overall and lower proportions of participants reported sub-optimal skills regarding lung cancer compared to thyroid cancer (see Fig. 1). Only 50% of pathologists in the UK agreed with the statement “I consider testing for RET altered cancers to be relevant to my practice”, compared to 90% in Germany, 67% in Japan, and 62% in the US (*p* = 0.634) (see Table 4).

**Table 3:** Pathologists who reported sub-optimal knowledge of select items by country (%, n)

	Country				
Knowledge item	**Germany (*n* = 20)**	**Japan (*n* = 30)**	**UK (*n* = 11)**	**USA (*n* = 30)**	**Total (*n* = 91)**
knowledge of genetic biomarker tests for thyroid cancers	30%, 6	79%, 23^a^	73%, 8	53%, 16	59%, 53
knowledge of genetic biomarker tests for lung cancers	15%, 3	60%, 18	55%, 6	30%, 9	40%, 36

^a^1 pathologist in Japan answered “Not relevant to my practice” for “knowledge of genetic biomarker tests for thyroid cancers”

**Table 4:** Pathologists’ responses (%, n) to agreement statements

**I consider ongoing trials on selective RET inhibitors useful in my practice**	**Germany (*n*** = **20)**	**Japan** **(*n*** **=** **22)**	**UK** **(*n*** **=** **5)**	**USA** **(*n*** **=** **28)**	**Total** **(*n*** **=** **75)**
Agree or strongly agree	85%, 17	32%, 7	60%, 3	32%, 9	48%, 36
Neither agree nor disagree	15%, 3	41%, 9	40%, 2	61%, 17	41%, 31
Disagree or strongly disagree	0%, 0	27%, 6	0%, 0	7%, 2	8%, 8
Not relevant to my practice^a^	0	8	6	2	16
**Relevant genetic biomarker tests for RET-altered thyroid cancer are available in my clinical setting**	**Germany (*n* = 20)**	**Japan (*n* = 26)**	**UK (*n* = 8)**	**USA (*n* = 30)**	**Total (*n* = 84)**
Agree or strongly agree	90%, 18	23%, 6	75%, 6	87%, 26	67%, 56
Neither agree nor disagree	5%, 1	8%, 2	13%, 1	10%, 3	8%, 7
Disagree or strongly disagree	5%, 1	69%, 18	13%, 1	3%, 1	25%, 21
Not relevant to my practice^a^	0	4	3	0	7
**I consider testing for RET altered cancers to be relevant to my practice**	**Germany (*n* = 20)**	**Japan (*n* = 27)**	**UK (*n* = 8)**	**USA (*n* = 29)**	**Total (*n* = 84)**
Agree or strongly agree	90%,18	67%,18	50%,4	62%,18	69%,58
Neither agree nor disagree	10%,2	26%,7	38%,3	31%,9	25%,21
Disagree or strongly disagree	0%,0	7%,2	13%,1	7%,2	6%,5
Not relevant to my practice^a^	0	1	0	1	2
**Relevant genetic biomarker tests for RET-altered lung cancer are available in my clinical setting**	**Germany (*n* = 20)**	**Japan (*n* = 28)**	**UK (*n* = 8)**	**USA (*n* = 30)**	**Total (*n* = 86)**
Agree or strongly agree	95%, 19	32%, 9	63%, 5	77%, 23	65%, 56
Neither agree nor disagree	5%, 1	21%, 6	13%, 1	17%, 5	15%, 13
Disagree or strongly disagree	0%, 0	46%, 13	25%, 2	7%, 2	20%, 17
Not relevant to my practice^a^	0	2	3	0	5
**The MDT approach is fully integrated in my clinical setting**	**Germany (*n* = 20)**	**Japan (*n* = 30)**	**UK (*n* = 11)**	**USA (*n* = 30)**	**Total (*n* = 91)**
Agree or strongly agree	95%, 19	47%, 14	82%, 9	97%, 29	78%, 71
Neither agree nor disagree	5%, 1	37%, 11	18%, 2	3%, 1	16%, 15
Disagree or strongly disagree	0%, 0	17%, 5	0%, 0	0%, 0	5%, 5
Not relevant to my practice^a^	0	0	0	0	0

^a^Participants who responded “Not relevant to my practice” were not included in the % of agreements

Pathologists in Japan and UK reported an overall lower level of skills performing specific biomarker tests, with biomarker tests for RET (*p* = 0.002) and NTRK (*p* =  < 0.001) alterations posing more of a challenge than tests for ALK (*p* = 0.01), EGFR and PD-L1 (both *p* =  < 0.001) alterations (see Table 5).

**Table 5:** %, (n) Pathologists who reported sub-optimal skills overall when *selecting or performing specific genomic biomarker tests*

*Sub-optimal skill level when performing test for:*	**Germany (*n* = 20)**	**Japan (*n* = 30)**	**UK (*n* = 11)**	**USA (*n* = 30)**
**ALK alteration**	20%, 4	63%, 19	45%, 5	30%, 9
**EGFR alteration**	15%, 3	67%, 20	45%, 5	26%, 8
**PD-L1 alteration**	10%, 2	67%, 20	45%, 5	33%, 10
**RET alteration**	30%, 6	82%, 23^a^	75%, 6^b^	50%, 15
**NTRK alteration**	25%, 5	86%, 26	72%, 8	62%, 18
*Sub -optimal skills when:*	**Germany (*n* = 20)**	**Japan (*n* = 29)**	**UK (*n* = 11)**	**USA (*n* = 30)**
Differentiating between RET-fusion and RET mutation based on test results^b^	15%,3	85%,23^a^	63%,5^b^	67%,20
Identifying relevant genomic biomarkers to inform the progression of **lung** cancer^a^	10%,2	72%,21	70%,7^c^	50%,15
Identifying relevant genomic biomarkers to inform the progression of **thyroid** cancer^a^	4%,8	8%,24	7%,8	6%,19

^a^2 pathologists in Japan answered, “Not relevant to my practice” for “RET alteration” and are not included in the percentages

^b^3 pathologists in UK answered, “Not relevant to my practice” for “RET alteration” and are not included in the percentages

^c^1 pathologist in UK answered, “Not relevant to my practice” for “lung cancer”

Alongside self-reported skill gaps, concerns were raised by numerous participants during interviews about the decision-making process regarding biopsies, skill level, and rigor of other health care providers who collect and analyse samples, depending on site and resources:

“*It depends on the degree of differentiation in the sample* […] *If you take a biopsy of a completely non-differentiated tumour* […] *and it is not clear whether or not the patient was really thoroughly examined beforehand or not, and so I must check whether this is a metastasis or a primary lung tumour.”*

- Pathologist, Germany

*“About 1/3 to 40% of histological and cytological pathology care in Germany is done by smaller practices, with 2-3 staff members -- We cannot expect that they are able to, on an interdisciplinary basis, be familiar with every mutation and every therapeutic algorithm.”*

- Pathologist, Germany

Overall lower confidence was reported in Japan and the US when interpreting genomic biomarker tests (ALK, EGFR, PD-L1 and RET); however, in all countries except Germany, confidence interpreting RET tests specifically was low when compared to other tests (see Table 6). In particular, the skills to differentiate between RET-fusion and RET mutation based on test results was sub-optimal in Japan (85%), UK (62%), and the US (67%) (*p* =  < 0.001) (Table 5).

**Table 6:** Pathologists’ confidence level in select items by country

Confidence item	**Country**	**Not relevant to my practice**	**N (who selected a value on the scale)**	**Mean**	**Std. Deviation**
Interpreting genetic biomarker tests – ALK	Germany	0	20	81.95	17.665
	Japan	1	29	65.31	26.734
	UK	0	11	63.55	35.859
	USA	1	29	69.00	27.717
Interpreting genetic biomarker tests—EGFR	Germany	0	20	80.75	19.687
	Japan	1	29	57.21	28.782
	UK	0	11	68.45	28.949
	USA	2	28	70.18	28.158
Interpreting genetic biomarker tests—PD-L1	Germany	0	20	84.35	13.048
	Japan	1	29	62.93	25.312
	UK	0	11	70.73	28.496
	USA	1	29	67.48	26.679
Interpreting genetic biomarker tests—RET	Germany	0	20	81.90	21.494
	Japan	5	25	47.44	25.333
	UK	3	8	37.13	32.529
	USA	2	28	59.29	29.867
Interpreting genetic biomarker test results from off-site laboratories	Germany	0	20	75.00	16.537
	Japan	3	27	58.26	27.208
	UK	0	11	59.18	34.336
	USA	1	29	70.69	27.172

Additionally, pathologists reported a gap in skills (*p* = 0.001) when selecting genomic biomarker tests to diagnose thyroid cancer in Japan (79%), the UK (73%), and the US (57%) (Fig. 1), and when identifying relevant genomic biomarkers to inform the progression of lung cancer (*p* = 0.009) (Japan – 72%, UK—70%, US—50%) in particular, when compared to thyroid cancer (Table 5). Qualitative data indicated that pathologists perceived these tests as either irrelevant, unreliable and/or in need of further validation:

*“We do not do* [testing panels] *because, ultimately, they tend to be risk markers and, so to speak, markers that one tests for in addition to thyroid diagnostics, that is ultimately very commercial and the relevance for the diagnosis and how the illness will proceed is questionable, therefore it is not included in the package of services which we offer for the thyroid.”*

- Pathologist, Germany

2) Challenges regarding communication and collaboration structures to support decision-making

Confusion about the structure of clinical decisions within a given healthcare setting was reported. In Japan, 73% of pathologists had sub-optimal knowledge of the responsibilities of tumour board members, compared to 45% in the UK, 25% in Germany, and 17% in the US (*p* =  < 0.001, see Table 7). This knowledge gap was also reported by endocrinologists (*p* =  < 0.001) in Japan (83%) and in the US (70%), as well as pulmonologists (*p* = 0.001) in Germany (34%) and Japan (64%). When asked to rate their knowledge of the type of information they should share with the tumour board, 80% of pathologists in Japan reported a gap, compared to 15% in Germany, 36% in the UK, and 13% in the US. This knowledge gap was also reported by medical oncologists in Germany (36%) and in the US (33%), endocrinologists in Germany (46%), Japan (85%), the UK (37%), and the US (50%) as well as pulmonologists in Japan (65%) (all *p* =  < 0.001, see Table 7).

**Table 7:** Sub-optimal knowledge of select items by specialty and country

		**Country**				
**Knowledge item**	**Specialty**	**Germany**	**Japan**	**UK**	**USA**	**Total**
The responsibilities of each member of the tumour board (*n* = 518)	Medical oncologist	28%, 12	-	28%, 12	24%, 11	27%, 35
	Pulmonologist	34%, 12	64%, 27	25%, 7	29%, 13	39%, 59
	Endocrinologist	46%, 12	83%, 35	46%, 19	70%, 26	55%, 92
	Pathologist	25%, 5	73%, 22	45%, 5	17%, 5	41%, 37
What type of information to share with the tumour board (*n* = 518)	Medical oncologist	36%, 16	-	23%, 10	33%, 14	33%, 40
	Pulmonologist	34%, 12	65%, 28	15%, 4	29%, 13	38%, 57
	Endocrinologist	46%, 12	86%, 36	37%, 15	50%, 19	56%, 82
	Pathologist	15%, 3	80%, 24	36%, 4	13%, 4	38%, 35

An endocrinologist described that there can sometimes be uncertainty about what information to share with multidisciplinary team members to ensure optimal patient-centered care:

*“My own view is pretty dim of most MDTs because often it is a big group of doctors, so an extremely expensive meeting, where it might be that nobody there has actually met the patient. Which means you don’t actually have the real story, or what the actual problem is, or you miss out […] So in our MDT we are very clear that one of us knows the story, knows the patient. It is patient-led rather than image-led or whatever, so that you try to make the right decisions for the individual, rather than just following endless protocols or pathways which may lead you down the wrong path.”*

- Endocrinologist, UK.

Additionally, collaboration across specialty areas was noticeably less common in Japan where only 47% of pathologists indicated that an MDT approach is used in their clinical setting as compared to Germany (95%), the UK (82%), and the US (97%) (*p* =  < 0.001). Insight into this finding was shared by a pathologist in Japan, describing that in their experience, meetings are infrequent, and communication is specialty-specific:

*“… we have meetings once every two months. I don’t participate in the pulmonology meetings, but I go to the head-neck meetings.”*

- Pathologist, Japan.

3) Perception of clinical trials for RET inhibitors

Only 32% of pathologists in Japan and 39% in the US agreed with the statement “I consider ongoing trials on selective RET inhibitors useful in my practice,” compared to 43% in the UK and 80% in Germany. Some pathologists expressed skepticism regarding how data collected in the controlled environment of a clinical trial can be applicable to their practice:

“*So, from the clinical trial data we can see, the molecular marker and the treatment results. But as, like, routine practice — that part of information actually is missing, and which is probably not right because a lot of trial data is in a more well-controlled environment. And so, whether the clinical trial data can actually be produced in a general practice — it’s probably still largely unknown.*”

- Pathologist, US

4) Barriers associated with governmental regulations

In Japan pathologists agreed that “relevant genomic biomarker tests for RET-altered lung (32%) and thyroid (23%) cancer are available in their clinical setting,” compared to 63% and 75% in the UK, 95% and 90% in Germany and 77% and 87% in the US (*p* =  < 0.001). According to interview data, government regulations on the use of approved companion diagnostics for a specific biomarker pose a prominent barrier to initiating optimal treatment in a timely manner based on test results. The issue of regulatory barriers was reported by participants from Japan in particular while integration of genomic testing into care pathways and widespread availability was reported to be an issue in the UK and the US.

“*Regarding lung cancer, the more sophisticated the examination the quicker we can receive all of the results and not limit the treatment to the companion diagnosis. Since the same gene mutation is found, there should be permission to give a broad interpretation, and the use of kinase inhibitors should be approved* […] *I feel like the progression of lung cancer is faster compared to other types of cancer. The patient’s cancer may move on to the next stage while we wait for all the tests, for BRAF, so I would like for those in charge of making the regulations to think about this topic deeply.”*

- Pathologist, Japan

## Discussion

Our data indicated that pathologists experience difficulties in key clinical areas and perceive that there are systems-level barriers to optimal care for patients with RET-altered cancers. Pathologists in this study reported that samples are often provided to them without all the relevant information they need. Some pathologists reported that their practices use off-site testing (Table 6), which posed challenges in terms of confidence in the results and requires coordinating access with sufficiently trained pathologists who have the specific skills needed for the management and performance of molecular profiling and biomarker testing and analysis. The smaller skill gaps for ALK and PDL-1 testing compared to those of RET testing (relatively less available in the US, UK, and Japan; see Table 3), suggest that intervening variables such as pathologist sub-speciality, which may determine their involvement in performing a particular type of test, may impact their familiarity and skills in that task. For example, PDL-1 testing can be performed by immunohistochemical methods with which most pathologists have much more familiarity, as opposed to the molecular methods required for RET analysis, generally the purview of molecular pathologists. Efforts to elevate skills should consider the required competencies of each pathology sub-specialisation, while emphasising collaboration.

The above barriers may slow the process of molecular testing and ultimately delay treatment initiation – concerns shared by study participants and documented in the literature [39]. Recently, Kerr et al. (2021) [39] reported country-specific differences in available resources but proposed that: 1) care should be guided by a multi-disciplinary tumour board, 2) reflex testing with expedited testing for known mutations should be implemented at diagnosis, and 3) there should be changes to biopsy practices that include tests such as NGS and rapid screening of liquid/solid biopsies in NSCLC to improve the chances of timely, accurate results. This study considered the broader testing considerations for both lung and thyroid cancers though challenges may arise at different points of care depending on type of thyroid cancer (medullary or differentiated). The results of this study and others indicate that optimal care must prioritise an in-depth understanding of these pathways, with timely testing for all actionable biomarkers in a given tumour type, inclusive of RET fusion in NSCLC and TC and RET mutations in MTC, adapted and adaptable to the patient profile. To ensure best practices, the current reliance on a limited number of specifically trained pathologists and advanced lab resources will need to be reimagined: a better distribution of human and technological resources will require multi-disciplinary and administrative collaboration to improve care on a health systems level [12].

A number of therapies for treating RET-altered lung and thyroid cancers have recently received FDA approval (e.g. selpercatinib and pralsetinib) and more are under investigation in several countries [39], including novel selective RET kinase inhibitors for medullary thyroid cancer that show promise in reducing the adverse effects associated with currently-used multikinase inhibitors [40]. Thus, the importance of RET testing can only have increased since the data were collected, further highlighting the need for relevant continuing medical education (CME) and continuing professional development (CPD) activities for pathologists.

These CME/CPD activities for pathologists should focus on the gaps identified by this study, starting with the selection and interpretation of the tests to be performed including sensitivity and specificity of the different tests available, and communicating interpreted results to clinicians in a way that optimally support their decision-making. These activities should also improve pathologists’ awareness of current clinical trials for RET inhibitors. National Medical Societies and other stakeholders in Education stakeholders could help address these gaps, through activities delivered at national congresses, such as workshops or Symposium, and other opportunities for peer-to-peer exchanges, such as interactive webinars or communities of practice.

Significant differences by country have been observed regarding the accessibility of genomic testing and targeted therapies [41]. This was a prominent issue for Japanese participants in this study. For Japan specifically, the unavailability of RET inhibitors and the absence of companion diagnostics for RET at the time of this study (a requirement for clinical testing in the country at the time of this study) may explain this finding. On an individual level, the financial burden of testing and treatment can be prohibitive to access. On a larger scale, much-needed efforts are underway to better coordinate medical systems, manufacturers, and regulators nationally and internationally to improve care for patients with RET-altered cancers [41]. It is important to note that in the time since data collection, specific RET inhibitors and companion diagnostics have become available in Japan.

The role of the pathologist may be more subspecialized in certain countries, either in terms of anatomic or molecular pathology, or by tumour site (i.e., thoracic pathology). Their subspecialty and consequently their different role, may have impacted their responses. However, our inclusion criteria required them to examine a minimum of 10 samples of lung or thyroid cancer a year, ensuring that their role included, at least minimally, the two targeted tumour sites.

Results of this study and others indicate that pathologists working with cancers that are commonly RET-altered (NSCLC and TC) would benefit from improvement to the core skills and confidence associated with their position, which may be obstructed by unfamiliarity with testing and a lack of consistent process for RET-testing in cancer. These include sample handling and testing practices, interpretation of test results, and multi- and inter-disciplinary educational interventions addressing the inefficiencies that occur at articulation points in the patient care pathway, including the transfer of samples off-site and communication with coordinated healthcare providers.

The data collection phase of this study coincided with the start of the COVID-19 pandemic, which disrupted aspects of testing and diagnosis practices, though this varied by country and cancer type [42–45]. Whether the adoption of remote technologies and changes to interdisciplinary collaboration as a result of the pandemic will have an effect on pathologists’ competencies or confidence is to be determined.

Prior to the pandemic, the treatment and testing landscape for RET altered cancers was rapidly evolving—much of the data collection in this study co-occurred with the approval process of RET inhibitors for use in lung and thyroid cancer, potentially limiting the participants’ awareness of the importance of testing for RET alterations.

Novel educational resources and early outreach may be beneficial and practical for pathologists, as pathologists in this study did not always consider clinical trial data useful to their practice, with the notable exception of Germany. This might be explained by the fact that the German Society of Pathology and the Federal Association of German Pathologists have an Independent Quality Assurance initiative which constantly update relevant information for the pathology community [46, 47]. Anticipated innovations and upcoming treatments could be a feature of a rigorously updated decision-making aid to assist pathologists in their role that also outlines the parameters of national regulations and reimbursement practices. Future studies that consider additional variables such as pathologist sub-specialty and the variations in their roles may provide greater insight into specific clinical challenges with the intention of improving education.

It is also important to address the collaboration challenges in lung and thyroid cancers and improve the integration of pathologists in the MDT to ensure effective transfer of information such as pathology reports. Collaboration competencies are increasingly required by professional bodies and have been highlighted by a number of competency models, such as the Accreditation Council for Graduate Medical Education (ACGME) core competencies [48] and the CanMEDS framework [49]. Evidence-based and competency-based CME/CPD are increasingly required by professional associations [50, 51].

### Limitations

This study aimed to explore and examine the experiences of pathologists in four countries whose health systems vary across and within national borders. As such, caution should be exercised when generalising the findings without regard to variables such as clinical setting or to an international scale. Purposive sampling (including participants with different years of practice and practice settings) was used to mitigate potential selection bias and to represent a range of pathologist perspectives pertaining to lung and thyroid cancers. Pathologist sub-specialty (e.g., anatomical pathologist, molecular pathologist) was not possible to consider as the sample size of this variable would be insufficient for meaningful analysis, though it is possible that sub-specialty may impact pathologists’ role, and therefore experiences, depending on their country.

Although sample sizes per country were relatively small, especially in the UK where recruitment was particularly challenging, combining qualitative and quantitative data helped increase the trustworthiness of the findings and the quantitative samples were large enough to allow for comparisons. Some survey items could not be divided by type of tumour (e.g., medullary or papillary TC) or RET-alteration (e.g., mutation or fusion) in order to keep the survey short.

## Conclusions

This mixed-method study identified multiple challenges faced by pathologists in their practice and highlighted educational needs for pathologists throughout the lung and thyroid cancer care pathways. The results of this study suggest that the reported knowledge and skills gaps, as well as reduced confidence in key areas of genomic biomarker testing and analysis for lung and thyroid cancers, are impacted by the availability and integration of comprehensive testing and targeted treatment. In countries such as Japan, as access to genomic testing improves, it is imperative that CME/CPD focus on increasing the readiness of pathologists to incorporate new practices.

Based on the findings of this study, educational initiatives should aim at increasing the knowledge of current guidelines and the strength of evidence for new tests and treatments. They should also aim at improving decision-making skills by creating clear decision pathways and decision-making tools to improve the confidence levels of the cancer care team, including considerations for crucial decision points along the care pathway. These findings are intended to inform national educational activities and offerings; however, more precise location-specific needs assessments would be a valuable area of further exploration to design relevant and impactful learning activities that benefit pathologists in their specific context.

## Data Availability

The datasets used and/or analysed during the current study are available from the corresponding author on reasonable request.

## Abbreviations

ALK: Anaplastic Lymphoma Kinase
CME: Continuing Medical Education
CPD: Continuing Professional Development
FISH: Fluorescence in Situ Hybridization
LC: Lung Cancer
MTC: Medullary Thyroid Cancer
NGS: Next Generation Sequencing
NSCLC: Non-Small Cell Lung Cancer
PDL1: Programmed Cell Death Ligand 1
RET: Rearranged During Transfection
TC: Thyroid Cancer
